# Application of a Novel Subject Classification Scheme for a Bibliographic Database Using a Data-Driven Correspondence

**DOI:** 10.3389/fdata.2019.00048

**Published:** 2020-01-09

**Authors:** Kei Kurakawa, Yuan Sun, Satoko Ando

**Affiliations:** ^1^Scholarly and Academic Information Division, Cyber Science Infrastructure Development Department, National Institute of Informatics, Tokyo, Japan; ^2^Information and Society Research Division, National Institute of Informatics, Tokyo, Japan; ^3^Clarivate Analytics (Japan), Co. Ltd., Tokyo, Japan

**Keywords:** bibliographic database, data-driven correspondence, research project database, subject classification scheme, topological space

## Abstract

A novel subject classification scheme should often be applied to a preclassified bibliographic database for the research evaluation task. Generally, adopting a new subject classification scheme is labor intensive and time consuming, and an effective and efficient approach is necessary. Hence, we propose an approach to apply a new subject classification scheme for a subject-classified database using a data-driven correspondence between the new and present ones. In this paper, we define a subject classification model of the bibliographic database comprising a topological space. Then, we show our approach based on this model, wherein forming a compact topological space is required for a novel subject classification scheme. To form the space, a correspondence between two subject classification schemes using a research project database is utilized as data. As a case study, we applied our approach to a practical example. It is a tool used as world proprietary benchmarking for research evaluation based on a citation database. We tried to add a novel subject classification of a research project database.

## Introduction

Subject classification is a popular and useful aspect for academic database and data analysis. Academic resources, such as research articles, journals, conference proceedings, books, field samples, software, and various electronic materials, are organized by subject classifications in the general or domain-specific approach. University libraries, institutional resource centers, and research labs organize their research resources in an efficient manner to gain easy access to these resources when necessary. Research funding agencies manage their applicants, projects, and reports by classifying research subjects, which are often diversified and transformed to reflect on the current research landscape. Academic fields are fundamental concepts of academic classifications for organizing academic materials. From analysis perspectives, institutional research (IR) focuses on research and educational activities, in which the research and educational portfolios of researchers, professors, and staff are analyzed through subject classifications. Moreover, the databases of national grants are often surveyed via subject classifications.

Practically, classification has been utilized in library catalogs for not less than a 100 years (Hjørland, 2008). The Dewey Decimal Classification is an old library classification invented in 1876 and is popular for classifying books in the shelves of university libraries. Other popular library classifications, such as the Universal Decimal Classification, the Library of Congress Classification, and the Colon Classification, were also invented a 100 years ago. They were revised several times to fit with the current book subject diversity. Japanese library classification examples include the Nippon Decimal Classification and the Japan National Diet Library Classification, which were released in 1928 and 1963, respectively. For academic journals, the Web of Science (WoS) subject classification is one of the most popular subject classifications for the WoS citation database. For research evaluation purposes, journals are frequently being classified based on specific viewpoints. The Essential Science Indicator (ESI) is one of the specifically developed subject classifications for research evaluation based on the WoS citation database.

In the research evaluation domain, special subject classifications that are developed for research and educational work should be adopted throughout all kinds of target databases (Gómez et al., 1996). National research and educational evaluation organizations use their original subject classifications to classify organizations and persons suitable for domestic evaluation tasks. Then, they compare them globally based on research and educational output records collected from world common output databases such as the WoS citation database. For example, the UK government defines Units of Assessment as subject classifications for the Research Assessment Exercise and the Research Excellence Framework. The Italian evaluation agency for university and research systems ANVUR (Agenzia Nazionale di Valutazione del Sistema Universitario e della Ricerca) constructed an original category scheme to be used for its evaluations. Excellence in Research for Australia, which is an Australian research evaluation program, developed the original subject classification scheme: Fields of Research. All these subject classifications must be adopted for the WoS citation database to analyze their national activities and compare them with regard to their common standards. Along with current international business qualifications on research evaluation, the same requirements emerged from the universities in Japan to ensure that the Japanese national funding programs KAKENHI subject classifications would be adopted for the WoS citation database.

However, adopting subject classifications for bibliographic databases is a highly challenging task. For example, in 2019, the WoS citation database in InCites™, which is a research output evaluation tool, comprised 58,395,008 article records of 24,688 journals. Even assigning subject categories for articles or journals as a set of units is labor intensive and time consuming. Hence, an excellent, effective, and efficient method for assigning their subject categories is necessary. In this study, we propose an approach to applying a novel subject classification scheme for the WoS citation database.

Our main contributions of the work are as follows:

- We propose an approach to apply a novel subject classification scheme for a subject-classified database using a data-driven correspondence between the new and present ones, which is accustomed to digital libraries.
- We give a fundamental analytical model of subject classification scheme based on set theory and describe compact topological space formation for a new subject classification scheme as a necessary condition.
- We demonstrate the effectiveness and efficiency of our approach to a practical bibliographic database.

In the following sections, firstly we look around the related work to state our approach in section Related Work, then describe our approach in the section Data-Driven Approach to Applying a Novel Subject Classification Scheme, and successively demonstrate the effectiveness and efficiency of our approach in a case study in section Case Study. Finally, we conclude our approach in the section Conclusions and Future Work.

## Related Work

In the above introductory section, we mentioned an issue around subject classifications from the viewpoint of library and information science and scientometrics. To tackle this issue, a series of related work in the computer science such as information retrieval, data mining, and digital libraries have been investigated for several decades. As for the general problem setting, subject classification of research items on bibliographic database refers to a part of automated text categorization problems. It goes back to Maron's (1961) seminal work on a probabilistic text classification. The methods are mainly divided into two types, i.e., supervised learning and unsupervised learning, which are also named as classification and clustering. The former requires the labeled data indicating the right answer to a given decision problem so as to derive classifiers. The classifiers then are applied to the target data to be classified. The example methods are naïve bays classification, neural networks, support vector machines. The latter does not need such the labeled data and extracts intrinsically the classification pattern from data and classify them. The example methods are k-means, expectation maximization (EM), hierarchical agglomerative clustering, divisive clustering, matrix decompositions, e.g., latent semantic indexing (LSI) and principal component analysis (PCA), and topic modeling, e.g., latent Dirichlet allocation (LDA). Sebastiani (Sebastiani, 2002) thoroughly surveyed these classifier techniques from the computer science perspective, and Jain et al. (1999) also surveyed clustering techniques for computer vision. Both classification and clustering are so general that they are frequently organized and explained from different basic contexts, such as pattern recognition (Bishop, 2006), information retrieval (Manning et al., 2008) and data mining (Han et al., 2011).

When adapting a method to the predefined classification scheme, classification is better utilized than clustering. Classification learns a decision from a labeled data, in contrast, clustering learns implicit relationships of unlabeled data. In relation to our problem setting, multi-label classification or multi-label learning have been investigated on several basic machine learning architectures. Multi-label classification classifies target data under 2^|*L*|^ classification space where *L* is a set of labels. Recent examples are multi-label learning based on SVM (Chang et al., 2017), based on deep learning (Mai et al., 2018), and based on ensemble classification (Büyükçakir et al., 2018). For very large classification space, extreme multi-label classification is proposed, e.g., a method based on graph embedding (Tagami, 2017), a method based on convolutional neural network (CNN) (Liu et al., 2017), and a method based on attention model of neural networks (Wang et al., 2018). Moreover, label hierarchy also can be considered so that part-of, is-a, and inclusion relationships are extracted from external data sources such as Wikipedia in the classification task (Bairi et al., 2016; Xie et al., 2017).

In digital libraries, the mappings between different classification schemes have been considered for a long period. For example, the method of automatically converting from existing classifications of documents into another scheme used in a quality-controlled database is occasionally used in co-operative cataloging projects and union catalogs, sometimes even in individual OPACs as soon as cataloging records using a different classification scheme are imported or exchanged (Koch et al., 1997). These mappings for the purpose of information integration and exchange was widely discussed in the 1970s, and is even more relevant today with the overall trend of information integration on the web (Slavic, 2011).

## Data-Driven Approach to Applying a Novel Subject Classification Scheme

Our approach is accustomed to digital libraries. We assume that a subject classification scheme has been originally adopted for a bibliographic database such as the WoS citation database. Then, we attempt to apply a new subject classification scheme for this database based on the relationship between two subject classification schemes. The relationship is the correspondence between them, which is induced by data.

### Subject Classification Model of the Bibliographic Database

First, we defined the subject classification model of the bibliographic database such as the WoS citation database to explain our approach. This model is a mathematical formula and a psychological aspect of subject categories embedded in the database.

Then, we assumed that a bibliographic database representing a set of articles for scientific research is available. Each article is labeled with at least one category of the subject classification scheme. That is, all articles are classified on the basis of the subject classification scheme. This scheme implies compact topological space in the database. It states the database structure that affects the analysis by using the subject classification scheme.

*Definition 1 (database with a subject classification scheme)*. A database *S* is a set of articles *a*_*n*_, and a subject classification scheme *C* is a set of subject categories *c*_λ_. Articles attributed to a subject category comprise a subset of *S*; hence, subject categories in a subject classification scheme refer to a family of subsets (*O*_λ_)_λ∈Λ_ of *S*. *O* is an open set, whereas Λ is an index set. A subset *O*_λ_ depends on the corresponding subject category *c*_λ_. Therefore, we define a map *f* from the subject classification scheme *C* to the powerset 𝔓(*S*).

*Theorem 1 (finite cover). A practical subject classification scheme* *C* *is mapped to a finite cover* 𝔒 *of* *S*.

*Proof*. In practical bibliographic databases, a subject classification scheme *C* consists of finite elements *c*_λ_ that are mapped to finite subsets *O*_λ_ using a map *f*. Let 𝔒 be a subset of 𝔓(*S*) comprising {*O*_*i*_|*i* ∈ *I*}, where *I* is a finite index set. Hence, *S* = ⋃_*i*∈*I*_ *O*_*i*_ (*O*_*i*_∈ 𝔒), where 𝔒 is referred to as a finite cover of *S*.

*Theorem 2 (compact topological space). A practical subject classification scheme* *C* *implies a compact topological space* $\left(S,\tilde{𝔒}\right)$.

*Proof*. In practical bibliographic databases, a subject classification scheme *C* consists of finite elements *c*_*i*_ that are mapped to finite subsets *O*_*i*_ using a map *f*. Let 𝔒 be a subset of 𝔓(*S*) comprising {*O*_*i*_|*i* ∈ *I*}, where *I* is a finite index set. For reference, let 𝔒_0_ be the subset of 𝔓(*S*) that consists of {∩_*i*∈*I*_*A*_*i*_|*A*_*i*_∈ 𝔒}, where the element is *S* if *I* = ∅. Let $\tilde{𝔒}$ be a subset of 𝔓(*S*) comprising {∪_λ∈Λ_*B*_λ_|*B*_λ_ ∈ 𝔒_0_}, where the element is ∅ if Λ = ∅. Here Λ is a finite or infinite index set. Thus, $\tilde{𝔒}⊃𝔒$, $S\in \tilde{𝔒}$, and $\emptyset \in \tilde{𝔒}$, where $\tilde{𝔒}$ is satisfied as a topology using the necessary and sufficient conditions. Theorem 1 also indicates a compact topological space $\left(S,\tilde{𝔒}\right)$. When a finite cover exists in a topological space, we refer to it as a compact topological space.

### Compact Topological Space Formation for a New Subject Classification Scheme

According to the subject classification model of the bibliographic database, we propose an approach of applying a new subject classification scheme for the database.

Here, we assume the following condition. A subject classification scheme *C*^(1)^ containing subject categories $c_{i}^{\left(1\right)}$ is mapped to a finite cover ${𝔒}^{\left(1\right)}=\left\{O_{i}^{\left(1\right)}|i\in I^{\left(1\right)}\right\}$ using a map *f*_1_, indicating a compact topological space $\left(S,{\tilde{𝔒}}^{\left(1\right)}\right)$.

Conventionally, we can use an approach to directly assign subject categories for the database records. We assign subject categories $c_{i}^{\left(2\right)}$ of a new classification scheme *C*^(2)^ to each article of *S*. Thus, a map *f*_2_ from *C*^(2)^ to a finite cover ${𝔒}^{\left(2\right)}=\left\{O_{i}^{\left(2\right)}|i\in I^{\left(2\right)}\right\}$ is constructed, implying a compact topological space $\left(S,{\tilde{𝔒}}^{\left(2\right)}\right)$.

In our approach, we develop a correspondence Γ : *C*^(2)^ → *C*^(1)^(Γ = (*C*^(2)^, *C*^(1)^; *G*), *G*⊂*C*^(2)^×*C*^(1)^), where $c_{i}^{\left(2\right)}\in C^{\left(2\right)},c_{j}^{\left(1\right)}$ $\in C^{\left(1\right)},\;c_{i}^{\left(2\right)}\times c_{j}^{\left(1\right)}\in G,C^{\left(2\right)}={⋃}_{i}\left\{c_{i}^{\left(2\right)}\right\}$, and $C^{\left(1\right)}={⋃}_{j}\left\{c_{j}^{\left(1\right)}\right\}$, to guarantee the existence of a finite cover.

Then, we construct a map

$$\begin{array}{l} g_{1}:C^{\left(2\right)}\to {\bar{ℭ}}^{\left(1\right)}\\ \;=\left\{{\bar{C}}_{i}^{\left(1\right)}|\begin{array}{c} \;c_{i}^{\left(2\right)}\in C^{\left(2\right)},\;c_{j}^{\left(1\right)}\in C^{\left(1\right)},\;c_{i}^{\left(2\right)}\times c_{j}^{\left(1\right)}\in G,\\ \;i\in I^{\left(2\right)},\;{\bar{C}}_{i}^{\left(1\right)}={⋃}_{j\in I_{i}^{\left(1\right)}}\left\{c_{j}^{\left(1\right)}\right\}\; \end{array}\;\right\}, \end{array}$$

where $S={⋃}_{i\in I^{\left(2\right)}}{\bar{C}}_{i}^{\left(1\right)},$ to be a finite cover. Finally, we establish a map

$$\begin{array}{l} g_{2}:{\bar{ℭ}}^{\left(1\right)}\to {\bar{𝔒}}^{\left(1\right)}\\ \;=\left\{{\bar{O}}_{i}^{\left(1\right)}|\begin{array}{c} {\bar{C}}_{i}^{\left(1\right)}\in {\bar{ℭ}}^{\left(1\right)},c_{j}^{\left(1\right)}\in {\bar{C}}_{i}^{\left(1\right)},O_{j}^{\left(1\right)}=f_{1}\left(c_{j}^{\left(1\right)}\right),\;\\ {\bar{O}}_{i}^{\left(1\right)}={⋃}_{j\in I_{i}^{\left(1\right)}}O_{j}^{\left(1\right)} \end{array}\right\}, \end{array}$$

where $S={⋃}_{i\in I^{\left(2\right)}}{\bar{O}}_{i}^{\left(1\right)},$ to be a finite cover. Hence, we obtain a composite map *g*_2_ ◦ *g*_1_ from *C*^(2)^ to a finite cover ${\bar{𝔒}}^{\left(1\right)}$, indicating a compact topological space $\left(S,{\tilde{\bar{𝔒}}}^{\left(1\right)}\right)$. Evidently, ${\tilde{\bar{𝔒}}}^{\left(1\right)}\subset {\tilde{𝔒}}^{\left(1\right)}$.

### Inducing a Correspondence Between Two Subject Classification Schemes Using a Research Project Database

To determine the correspondence between two subject classification schemes, experts of the subject classification schemes normally discuss the relationship structure of these schemes based on their knowledge and practical experiences.

In our approach, the actors are data scientists who analyze a database wherein an entity is categorized into two subject classification schemes and then induce the correspondence between them through an analysis.

As evidence data, anything that includes information of the relationship between the two subject classification schemes is useful. One of the available resources is a research project database that is rather popular among academic databases. In our case, it is the research project database KAKEN that includes the structural relationship between the WoS and KAKENHI subject categories. Thus, we ensure that our approach can adopt the research project database.

#### Using a Research Project Database

We define a research project database such as the KAKEN database as follows. A research project database *T* describes research projects *b*_*n*_, one of whose outputs is a list of research articles *a*_*n*_ on a bibliographic database *S*.

The research articles *a*_*n*_ of *S* are categorized with a subject classification scheme *C*^(1)^. We define the map *f*_1_ by which *C*^(1)^ is mapped to a finite cover ${𝔒}_{S}^{\left(1\right)}=\left\{O_{i}^{\left(1\right)}|i\in I^{\left(1\right)}\right\}$ of *S*, implying a compact topological space $\left(S,{\tilde{{𝔒}_{S}}}^{\left(1\right)}\;\right)$.

The research projects *b*_*n*_ of *T* are categorized with a subject classification scheme *C*^(2)^. We define a map *h*_1_ by which *C*^(2)^ is mapped to a finite cover ${𝔒}_{T}^{\left(2\right)}=\left\{O_{i}^{\left(2\right)}|i\in I^{\left(2\right)}\right\}$ of *T*, implying a compact topological space $\left(T,{\tilde{{𝔒}_{T}}}^{\left(2\right)}\;\right)$.

We define a map *h*_2_:*T* → 𝔓(*S*) to ensure that a research project produces a set of research articles. Here, let the image of the map be reduced to 𝔖 (⊂𝔓(*S*)) to become a surjection. Then, we also define a map $h_{2}\prime :T\to P\left(S\prime \right)$, where $S^{\prime }={⋃}_{i\in I_{𝔖}}O_{i}\left(O_{i}\in 𝔖\right)$ and *S*′ ⊂ *S*. For the image *S*′, we define a map *f*_1_′ by which *C*^(1)^ is mapped to a finite cover $O_{S\prime }^{\left(1\right)}=\left\{O_{i}^{\prime \left(1\right)}|i\in I^{\left(1\right)}\right\}$ of *S*′, implying a compact topological space $\left(S\prime ,{\tilde{O_{S\prime }}}^{\left(1\right)}\right)$.

Next, we develop a map

$$\begin{array}{l} h_{3}:{𝔒}_{T}^{\left(2\right)}\to O_{S\prime }^{\left(2\right)}\\ \;=\{{\bar{O}}_{S\prime }^{\left(2\right)}|\begin{array}{c} \begin{array}{c} O_{Ti}^{\left(2\right)}\in O_{T}^{\left(2\right)},b_{j}^{\left(2\right)}\in O_{Ti}^{\left(2\right)},\;O_{S\prime j}^{\left(2\right)}=h_{2}\prime (b_{j}^{\left(2\right)}),\\ {\bar{O}}_{S\prime i}^{\left(2\right)}=\cup_{j}O_{S\prime j}^{\left(2\right)}, \end{array} \end{array}\} \end{array}$$

which is a subset of 𝔓(*S*′), where ${𝔒}_{S^{\prime }}^{\left(2\right)}$ is a finite cover. Subsequently, we obtain a composite map $h_{3}◦h_{1}:C^{\left(2\right)}\to {𝔒}_{S^{\prime }}^{\left(2\right)}$. Considering that ${𝔒}_{S^{\prime }}^{\left(2\right)}$ is a finite cover, it induces a compact topological space.

In this case, we validated the following robust suppositions. The composite map $h_{3}◦h_{1}:C^{\left(2\right)}\to {𝔒}_{S^{\prime }}^{\left(2\right)}$ represents the classification of articles using the subject classification scheme. Moreover, if two images on *S*′ of maps *f*_1_′ and *h*_3_ ◦ *h*_1_ are equivalent, then their inverse images also have the same relation.

#### Natural Overlapping Between Two Subject Classification Schemes

Here, we obtained actual data on the relationship between two subject classification schemes on a database. We have a database *S*′ and two sets of finite covers ${𝔒}_{S^{\prime }}^{\left(1\right)}$ and ${𝔒}_{S^{\prime }}^{\left(2\right)}$ that are images from *C*^(1)^ and *C*^(2)^.

In natural phenomena, we often observe statistical laws of nature. A popular law in the linguistic field, that is, Zipf's law, states that the frequency of words follows a distribution where the word rank *n* has a frequency proportional to 1/*n*. Generally, the same distribution is observed in natural phenomena, referred to as a power law, which is denoted by ln *p*(*x*) = −α ln *x* + *c*, where α and *c* are constants (Newman, 2005). For example, all the following obey power law distributions: the sizes of city populations, earthquakes, moon craters, solar flares, computer files, and wars; the occurrence frequency of personal names in most cultures; the number of papers written by scientists; the number of citations received by papers; the number of hits on web pages; and the sales of books, music recordings, and almost every other branded commodity.

When actual data are analyzed, the power law trend in most cases holds only for an intermediate range of values; a power law breakdown exists in the distribution tails (Martínez-Mekler et al., 2009). The reason for this is finite size effects (e.g., insufficient data for good statistics), network dilution, network growth constraints, and different underlying dynamical regimes. Thus, power law corrections (sometimes referred to as scaling corrections) occur in the form of exponential, Gaussian, stretched exponential, gamma, and various types of extreme value distributions. This phenomenon obeys a discrete version of a generalized beta distribution, which is given by *f*(*r*) = (*A*(*N* + 1 − *r*)^*b*^)/*r*^*a*^. Here, *r* is the rank, *N* is its maximum value, *A* denotes the normalization constant, and (*a, b*) are two fitting exponents.

In our case, the elements of finite covers ${𝔒}_{S^{\prime }}^{\left(1\right)}$ and ${𝔒}_{S^{\prime }}^{\left(2\right)}$ represent natural overlapping sets. For $O^{\left(2\right)}(\in \;O_{S^{\prime }}^{\left(2\right)})$), its intersections *O*^(2)^ ∩ *O*^(1)^ to all $O^{\left(1\right)}(\in \;O_{S^{\prime }}^{\left(1\right)}\;)$) are present. Its cardinalities greater than zero, if sorted in rank order, obey the discrete version of the generalized beta distribution given that the subject categories are finite.

#### Metrics for Inducing a Correspondence

To identify a correspondence between *C*^(1)^ and *C*^(2)^, we attempt to find a subset $\left\{O_{i}^{\left(1\right)}|i\in I_{j}^{\left(1\right)}\right\}$ of $O_{S\prime }^{\left(1\right)}$ for $O_{j\in I^{\left(2\right)}}^{\left(2\right)}$ to be ideally satisfied that $O_{j}^{\left(2\right)}={⋃}_{i\in I_{j}^{\left(1\right)}}O_{i}^{\left(1\right)}.$ However, in most cases, $O_{j}^{\left(2\right)}⊅O_{i}^{\left(1\right)}$ and $O_{j}^{\left(2\right)}\neq {⋃O}_{i}^{\left(1\right)}$. Hence, we first define the following metrics: (precision)

$$\begin{array}{l} d_{pj}=\frac{\left|{⋃}_{i\in I_{j}^{\left(1\right)}}\left(O_{j}^{\left(2\right)}\cap O_{i}^{\left(1\right)}\right)\right|}{\left|{⋃}_{i\in I_{j}^{\left(1\right)}}O_{i}^{\left(1\right)}\right|} \end{array}$$

and (recall)

$$\begin{array}{l} d_{rj}=\frac{\left|{⋃}_{i\in I_{j}^{\left(1\right)}}\left(O_{j}^{\left(2\right)}\cap O_{i}^{\left(1\right)}\right)\right|}{\left|O_{j}^{\left(2\right)}\right|}. \end{array}$$

Then, we define the generalized harmonic mean of precision and recall: (*F*_β_-measure)

$$\begin{array}{l} d_{fj}=\frac{\left(1+\beta^{2}\right)d_{pj}d_{rj}}{\beta^{2}d_{pj}+d_{rj}},\;\beta >0. \end{array}$$

Finally, we use the *F*_β_-measure to determine which element has a correspondence relation. The basic strategy is to choose the subset $\left\{O_{i}^{\left(1\right)}|i\in I_{j}^{\left(1\right)}\right\}$ which maximize the *F*_β_-measure. β affects the weight balance between *d*_*pj*_ and *d*_*rj*_ for *d*_*fj*_. β = 1 indicates the equivalent balance between them. We can use the β to control the balance in relation to the existence of a finite cover.

In practical cases, we might project the cardinal number of the subsets onto the contingency table between two subject classification schemes. A contingency table, or a two-way frequency table, is a tabular mechanism with rows and columns used in statistics to present categorical data in terms of frequency counts.

By using the contingency table that represents the overall counting of elements, we calculate the following pseudo precision, recall, and *F*_β_-measure based on the original definitions:(pseudo precision)

$$\begin{array}{l} d_{pj}^{\prime }=\frac{\sum_{i}|O_{j}^{\left(2\right)}\cap O_{i}^{\left(1\right)}|}{\sum_{i}|O_{i}^{\left(1\right)}|} \end{array}$$

and (pseudo recall)

$$\begin{array}{l} d_{rj}^{\prime }=\frac{\sum_{i}|O_{j}^{\left(2\right)}\cap O_{i}^{\left(1\right)}|}{\left|O_{j}^{\left(2\right)}\right|}. \end{array}$$

Then, the generalized harmonic mean of precision and recall is also calculated: (pseudo *F*_β_-measure)

$$\begin{array}{l} d_{fj}^{\prime }=\frac{\left(1+\beta^{2}\right)d_{pj}^{\prime }d_{rj}^{\prime }}{\beta^{2}d_{pj}^{\prime }+d_{rj}^{\prime }},\;\beta >\;0. \end{array}$$

The values of precision and pseudo precision, the values of recall and pseudo recall, and the values of *F*_β_-measure and pseudo *F*_β_-measure can be different because of subadditivity.

#### The Main Steps to Work on Our Approach

**Figure 1:**
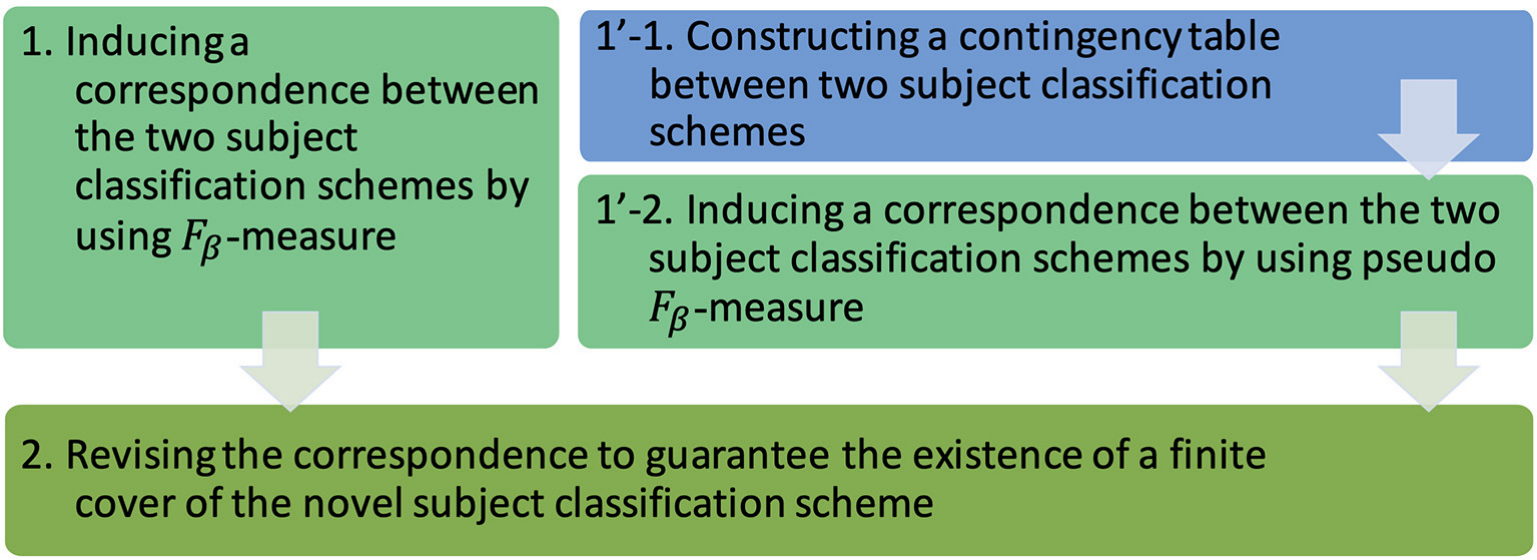
The main steps to work on our methodology: constructing a contingency table, inducing a correspondence, and revising the correspondence.

To follow the methodology above, the main steps of the work can be illustrated in Figure 1. The first step is to induce a correspondence between two subject classification schemes by using *F*_β_-measure (step 1 in the figure). In practical cases, alternatively, the first step is to construct a contingency table between two subject classification schemes (step 1'-1) and then induce a correspondence between them by using pseudo *F*_β_-measure (step 1'-2). The second step is to revise the correspondence to guarantee the existence of a finite cover of the novel subject classification scheme (step 2).

## Case Study

To verify our approach described previously, we adapt it for a practical case. A world-leading research output evaluation tool, that is, InCites™, which is produced by Clarivate Analytics, Co., Ltd., provides bibliometric analysis functions, wherein bibliometrics can be analyzed using domestic, WoS, and ESI subject classification schemes. Japanese users are eager to utilize the subject classification scheme of Japan's largest national research grants KAKENHI to analyze their IR outputs on the system. The WoS citation database comprises bibliographic records originally classified using the WoS subject classification scheme. The KAKENHI subject classification scheme is a novel subject classification scheme to be applied in the WoS citation database. We were occasionally given an opportunity to deal with this challenging task.

### Inducing a Correspondence Between the WoS and KAKENHI Subject Categories

We use the following steps to induce a correspondence between the WoS and KAKENHI subject categories.

#### Developing a Contingency Table as Evidence Data

We construct a contingency table between the WoS and KAKENHI subject categories to induce a correspondence.

The research project database KAKEN represents the archival records of research projects and the outputs of KAKENHI grants in Japan. The KAKEN database contains the descriptions of projects started after 1964 and the lists of their outputs, including journal articles, conference proceedings, reports, and books. The research projects are classified using the KAKENHI subject classification scheme that has been defined for the corresponding year.

In this study, we select the research projects in 2009 whose KAKENHI subject classification scheme consists of a hierarchical structure: four categories, 10 areas, 67 disciplines, and 284 research fields. The total number of projects is 58,952, and that of output publications that might be written in English is 173,940.

**Figure 2:**
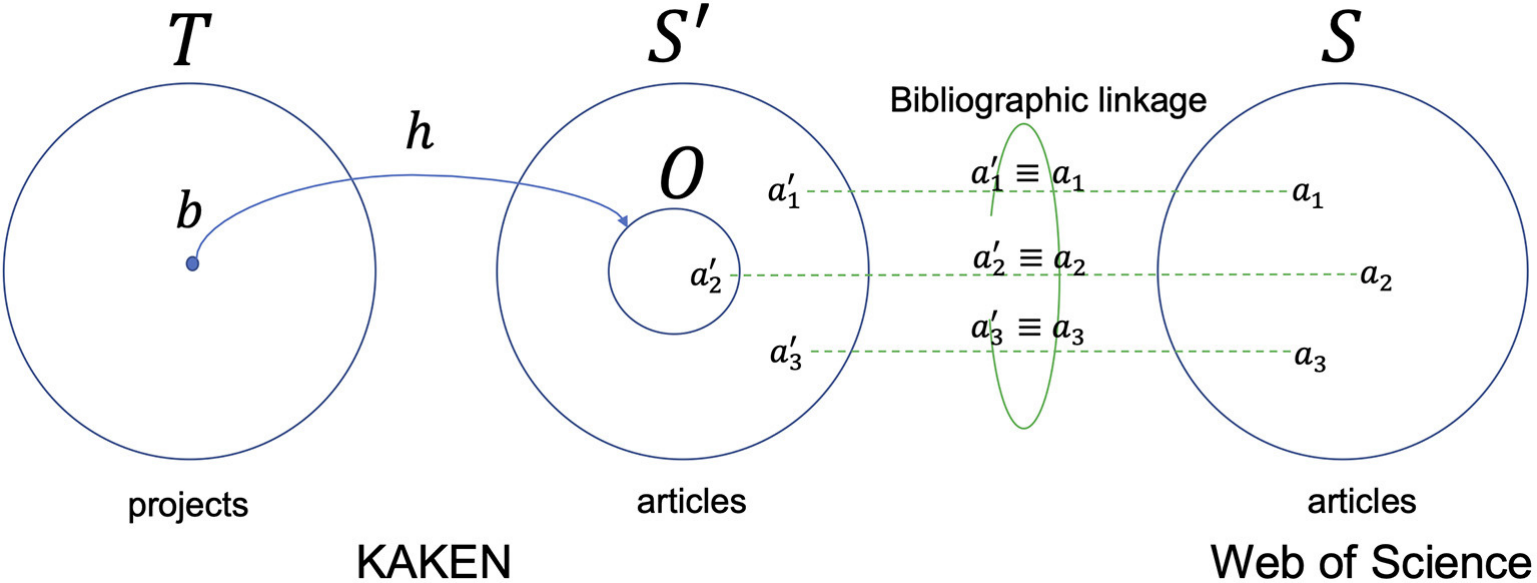
Bibliographic linkage between the KAKEN and WoS citation databases in a Venn diagram.

The English articles in the KAKEN database are listed in a citation format, and it is not yet clear to which WoS categories they are assigned. Hence, we identified the same bibliographic records in the WoS citation database as of 2009 and 2010 (99.8% of output publications are published in the years) through a set of record linkage techniques to obtain a set of articles *S*′ that are classified using both the KAKENHI and WoS classification schemes, as depicted in Figure 2 (Kurakawa et al., 2014). The size of the adopted WoS citation database was 3,925,776, which is classified with 251 subject categories of the WoS classification scheme and 22 subject categories of the ESI classification scheme.

Consequently, we obtained a total of 75,042 pairs of citations, which is 43.1% of the 173,940 articles listed in the KAKEN database. The record linkage technique uses i-Linkage (Aizawa and Oyama, 2005) as a ranking function and SVM as a classification function to identify the same bibliographic records in the KAKEN database and the WoS citation database. In a 10-fold cross validation of 800 samples, the accuracy of the linkage was 0.9501. The precision, recall, and f-measure were 0.9492, 0.9510, and 0.9498, respectively.

**Figure 3:**
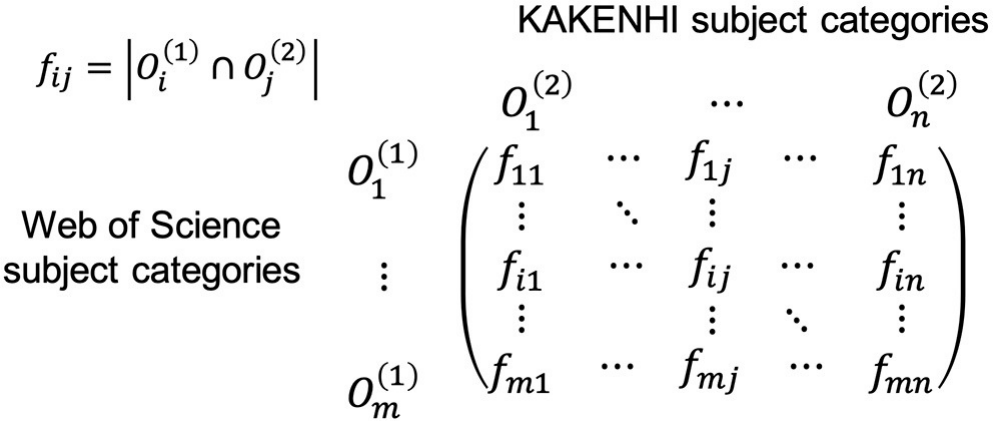
Contingency table for the KAKENHI and WoS subject categories.

**Figure 4:**
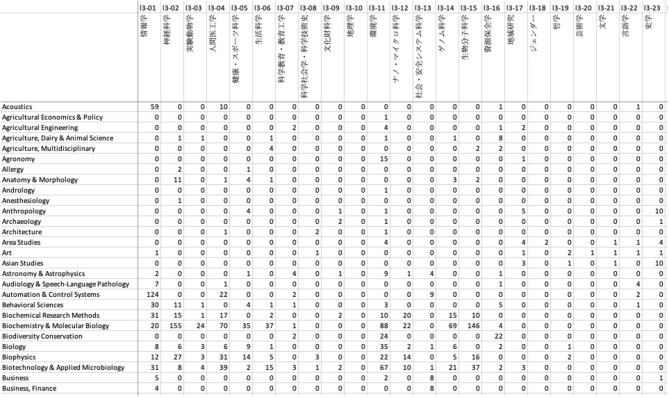
Example screen of Excel showing part of the contingency table between the third-level subject categories of the KAKENHI and WoS subject classification schemes.

We next constructed a contingency table for the two subject classification schemes based on this linkage result, as illustrated in Figure 3. An example in Figure 4 shows part of the contingency table between the third-level 67 KAKENHI and 251 WoS subject categories.

Among the 75,042 pairs of citations, those categorized with both the subject classification schemes were reduced to 59,595 pairs because the 52,956 out of the total 58,952 research projects are assigned with the KAKENHI subject classification scheme and the others are not.

When the overall counting of the citations to each subject category was applied, we obtained the sum of 97,175 frequency counts in the contingency table. In the WoS citation database, each article is assigned one or more subject categories of the WoS classification scheme, and is simultaneously assigned one subject category of the ESI classification scheme. When we count a citation assigned to multiple subject categories, the frequency count is increased by one for each corresponding subject category. In the KAKEN database, each article is assigned one subject category of the KAKENHI classification scheme, the frequency count is increased by one for the corresponding subject category. Thus, for a citation, the frequency counts in the contingency table are increased by the number of WoS categories or ESI categories, and it looks like many articles were published under the corresponding KAKENHI category.

#### Analysis of the Contingency Table

To clearly show what happens in the contingency table, we compared the distribution among the WoS subject categories with that of the KAKENHI subject category. We observed a good fit of the discrete generalized beta distribution to the rank-ordering distribution in the contingency table.

**Figure 5:**
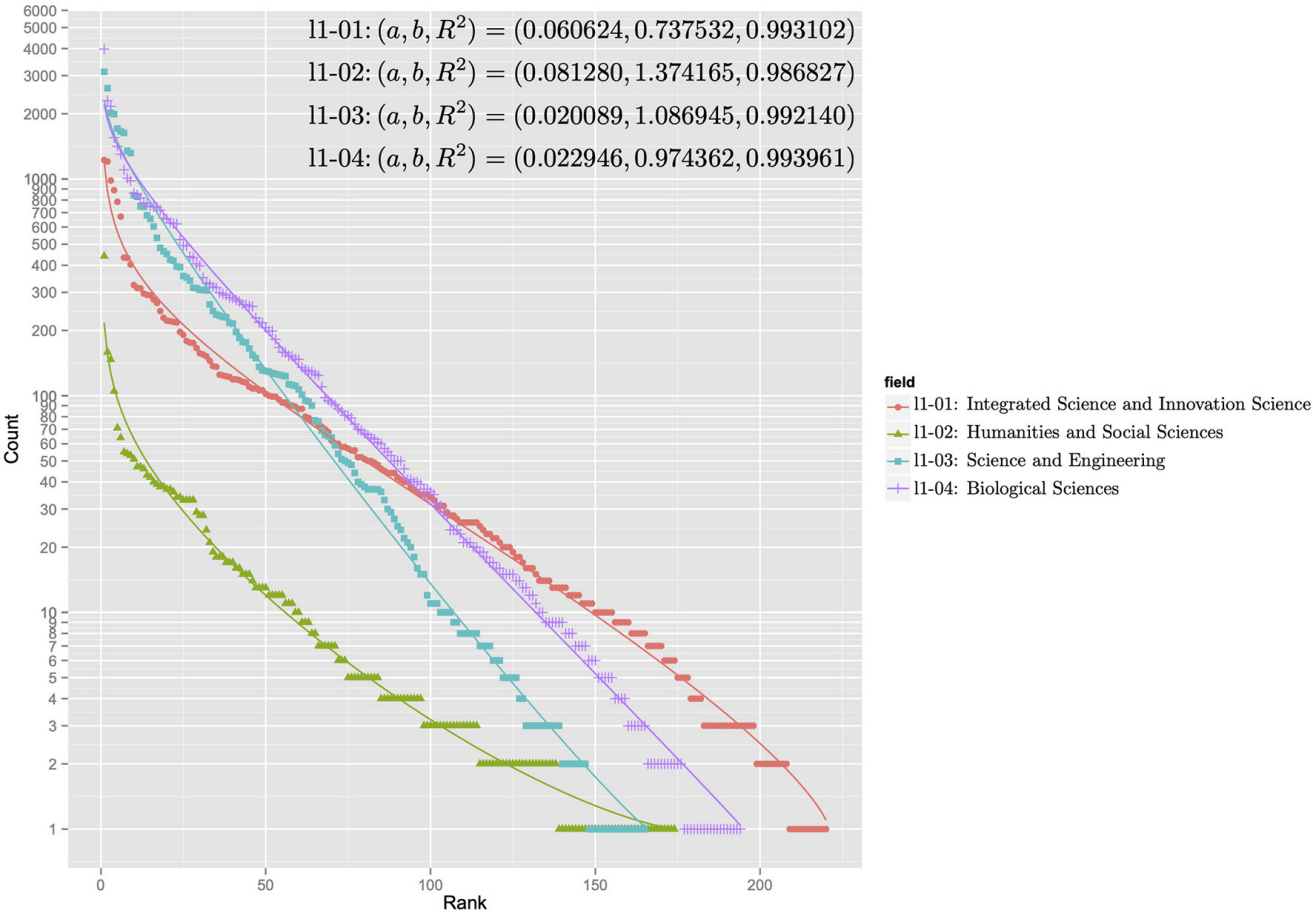
Rank-ordering distributions for the first-level subject categories of the KAKENHI subject classification scheme.

**Figure 6:**
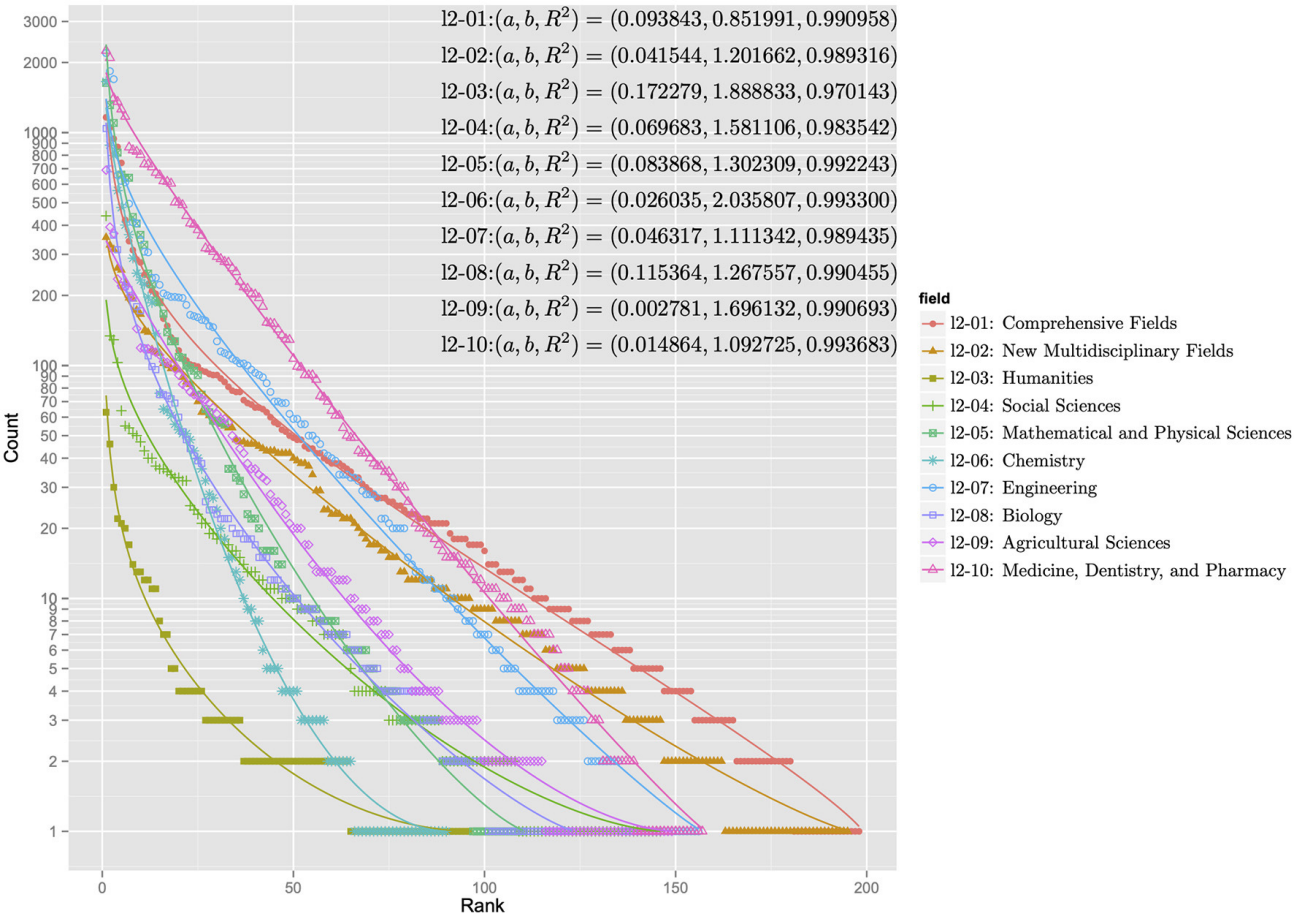
Rank-ordering distributions for the second-level subject categories of the KAKENHI subject classification scheme.

Figures 5, 6 show the rank-ordering distributions for the first and second levels of the subject categories of the KAKENHI subject classification scheme. The first-level subject categories include “Integrated Science and Innovative Science” (l1-01), “Humanities and Social Sciences” (l1-02), “Science and Engineering” (l1-03), and “Biological Sciences” (l1-04). The second-level subject categories include “Comprehensive Fields” (l2-01), “New Multidisciplinary Fields” (l2-02), “Humanities” (l2-03), “Social Sciences” (l2-04), “Mathematical and Physical Sciences” (l2-05), “Chemistry” (l2-06), “Engineering” (l2-07), “Biology” (l2-08), “Agricultural Sciences” (l2-09), and “Medicine, Dentistry, and Pharmacy” (l2-10). For each KAKENHI subject category at any level, the frequencies corresponding to the 251 WoS subject categories are sorted in rank order. If the frequency is zero, then the WoS subject category is omitted in the distribution. The *x* axis of the graph represents the rank, and the *y* axis of the graph denotes the log scale of the frequency count. With these scales, the discrete generalized beta distribution is fitted to the data to ensure that R-squared as a goodness-of-fit statistical score ranges from 0.986 to 0.994 for the first level and from 0.970 to 0.994 for the second level. In this case, the sets of parameters *a* and *b* that affect the figures of the distribution vary.

The distributions in the graph can be categorized into two types: concentration and dispersal. In the first level of the KAKENHI subject categories, the concentration type refers to the graph of “Science and Engineering” (l1-03) and “Biological Sciences” (l1-04). The dispersal type refers to the graph of “Integrated Science and Innovative Science” (l1-01). In the second level, the concentration type refers to the graph of “Humanities” (l2-03), “Chemistry” (l2-06), and “Mathematical and Physical Sciences” (l2-05). The dispersal type refers to the graph of “Comprehensive Fields” (l2-01) and “New Multidisciplinary Fields” (l2-02). The concentration type means the subject category is a specialized field. The dispersal type means the subject category is a multidisciplinary field.

For all the distributions, the goodness of fit to the discrete generalized beta distribution implies that a set of articles categorized to the KAKENHI subject category naturally overlaps that of articles categorized to the WoS subject categories at any level. However, the overlapping degree depends on the target subject categories.

#### Maximizing the F-Measure

Here, we analyzed the possibility that each KAKENHI subject category overlaps with the WoS subject categories. The aim of inducing a correspondence between the KAKENHI and WoS subject categories encouraged us to calculate the *F*_β_-measures between these subject categories.

Table 1 lists the maximum pseudo *F*_1_-measure, and the corresponding precision, and recall to produce the maximum pseudo *F*_1_-measure, the number of WoS subject categories to get the maximum pseudo *F*_1_-measure, and the cardinality for the third-level 67 disciplines of the KAKENHI subject classification scheme. The order of the disciplines in the list is the same as that of the KAKENHI subject classification scheme. The disciplines which are under the same area are listed together as a group. Each 67 discipline is included in either of 10 areas. For example, “Informatics” (l3-01), “Brain Sciences” (l3-02), “Laboratory Animal Science” (l3-03), “Human Informatics” (l3-04), “Health/Sports Science” (l3-05), “Human Life Science” (l3-06), “Science Education/Educational Technology” (l3-07), “Sociology/History of Science and Technology” (l3-08), “Cultural Assets Study” (l3-09), “Geography” (l3-10) are under the same area “Comprehensive Fields” (l2-01). In the same way, “Environmental Science” (l3-11) to “Gender” (l3-18) are under “New Multidisciplinary Fields” (l2-02), “Philosophy” (l3-19) to “Cultural Anthropology” (l3-25) are under “Humanities” (l2-03), “Law” (l3-26) to “Education” (l3-32) are under “Social Sciences” (l2-04), “Mathematics” (l3-33) to “Plasma Science” (l3-37) are under “Mathematical and Physical Sciences” (l2-05), “Basic Chemistry” (l3-38) to “Materials Chemistry” (l3-40) are under “Chemistry” (l2-06), “Applied Physics” (l3-41) to “Integrated Engineering” (l3-48) are under “Engineering” (l2-07), “Basic Biology” (l3-49) to “Anthropology” (l3-51) are under “Biology” (l2-08), “Plant Production and Environmental Agriculture” (l3-52) to “Boundary Agriculture” (l3-59) are under “Agricultural Sciences” (l2-09), “Pharmacy” (l3-60) to “Nursing” (l3-67) are under “Medicine, Dentistry, and Pharmacy” (l2-10).

**Table 1:** Maximum pseudo F_1_-measure for the third-level 67 disciplines of the KAKENHI subject categories against the 251 WoS subject categories.

**The third-level 67 disciplines seq. no**.	**KAKENHI subject category**	**Translation**	**Cardinality**	**No. of WoS subject categories to get the max pseudo F_**1**_-measure**	**Pseudo precision**	**Pseudo recall**	**Max. pseudo F_**1**_-measure**
(l3-01)	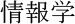	Informatics	6,637	17	0.576	0.626	0.600
(l3-02)	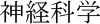	Brain sciences	1,570	1	0.218	0.365	0.273
(l3-03)	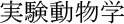	Laboratory animal science	242	1	0.059	0.074	0.066
(l3-04)	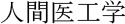	Human informatics	1,995	8	0.222	0.213	0.217
(l3-05)	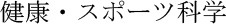	Health/sports science	844	5	0.181	0.290	0.223
(l3-06)	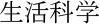	Human life science	467	4	0.239	0.281	0.258
(l3-07)	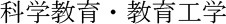	Science education/educational technology	388	2	0.377	0.103	0.162
(l3-08)	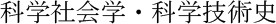	Sociology/history of science and technology	43	6	0.111	0.163	0.132
(l3-09)	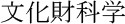	Cultural assets study	55	1	0.200	0.036	0.062
(l3-10)	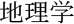	Geography	148	4	0.117	0.203	0.149
(l3-11)	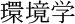	Environmental science	2,136	14	0.262	0.385	0.312
(l3-12)	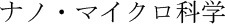	Nano/micro science	1,852	4	0.103	0.313	0.155
(l3-13)	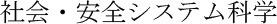	Social/safety system science	868	14	0.187	0.214	0.199
(l3-14)	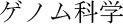	Genome science	394	3	0.040	0.203	0.067
(l3-15)	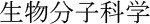	Biomedical engineering	875	2	0.119	0.325	0.174
(l3-16)	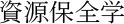	Culture assets and museology	172	3	0.181	0.145	0.161
(l3-17)	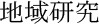	Area studies	85	7	0.164	0.271	0.204
(l3-18)	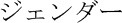	Gender	27	3	0.231	0.111	0.150
(l3-19)	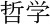	Philosophy	60	4	0.436	0.283	0.343
(l3-20)	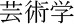	Art Studies	9	1	0.091	0.111	0.100
(l3-21)	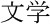	Literature	41	10	0.700	0.683	0.691
(l3-22)	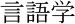	Linguistics	239	3	0.705	0.410	0.519
(l3-23)	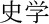	History	82	6	0.412	0.341	0.373
(l3-24)	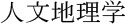	Human geography	14	3	0.175	0.500	0.259
(l3-25)	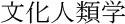	Cultural anthropology	38	3	0.056	0.105	0.073
(l3-26)	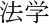	Law	41	3	0.385	0.122	0.185
(l3-27)	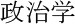	Politics	59	2	0.409	0.458	0.432
(l3-28)	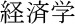	Economics	992	12	0.692	0.622	0.655
(l3-29)	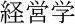	Management	130	5	0.294	0.385	0.333
(l3-30)	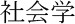	Sociology	90	8	0.176	0.278	0.216
(l3-31)	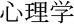	Psychology	794	14	0.488	0.479	0.483
(l3-32)	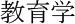	Education	151	9	0.244	0.258	0.251
(l3-33)	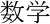	Mathematics	2,589	4	0.734	0.792	0.762
(l3-34)	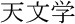	Astronomy	1,005	1	0.505	0.870	0.639
(l3-35)	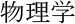	Physics	5,199	6	0.498	0.651	0.565
(l3-36)	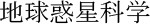	Earth and planetary science	2,099	7	0.619	0.662	0.640
(l3-37)	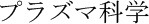	Plasma science	508	1	0.233	0.191	0.210
(l3-38)	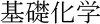	Basic chemistry	2,448	7	0.229	0.801	0.356
(l3-39)	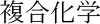	Applied chemistry	3,573	6	0.283	0.526	0.368
(l3-40)	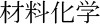	Materials chemistry	1,635	7	0.157	0.348	0.216
(l3-41)	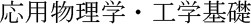	Applied physics	2,235	5	0.170	0.394	0.238
(l3-42)	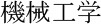	Mechanical engineering	2,675	11	0.431	0.388	0.408
(l3-43)	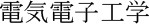	Electrical and electric engineering	4,875	10	0.338	0.669	0.449
(l3-44)	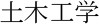	Civil engineering	711	8	0.371	0.484	0.420
(l3-45)	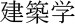	Architecture and building engineering	170	3	0.286	0.506	0.365
(l3-46)	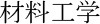	Material engineering	2,931	6	0.348	0.523	0.418
(l3-47)	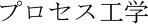	Process/chemical engineering	1,283	4	0.145	0.306	0.197
(l3-48)	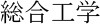	Integrated engineering	1,465	8	0.256	0.309	0.280
(l3-49)	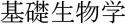	Basic biology	2,423	7	0.375	0.400	0.387
(l3-50)	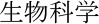	Biological science	2,679	4	0.167	0.582	0.259
(l3-51)	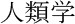	Anthropology	300	3	0.315	0.440	0.367
(l3-52)	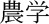	Plant production and environmental agriculture	899	4	0.307	0.449	0.365
(l3-53)	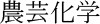	Agricultural chemistry	1,755	6	0.220	0.386	0.281
(l3-54)	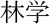	Forest and forest products science	559	5	0.408	0.252	0.312
(l3-55)	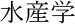	Applied aquatic science	581	2	0.419	0.327	0.367
(l3-56)	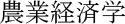	Agricultural science in society and economy	31	2	0.333	0.097	0.150
(l3-57)	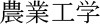	Agro-engineering	216	4	0.157	0.259	0.195
(l3-58)	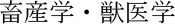	Animal life science	1,190	4	0.511	0.387	0.440
(l3-59)	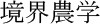	Boundary agriculture	541	4	0.235	0.148	0.181
(l3-60)	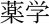	Pharmacy	3,457	4	0.294	0.369	0.328
(l3-61)	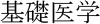	Basic medicine	5,232	16	0.213	0.551	0.307
(l3-62)	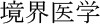	Boundary medicine	850	12	0.162	0.112	0.132
(l3-63)	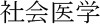	Society medicine	1,065	8	0.282	0.262	0.271
(l3-64)	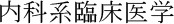	Clinical internal medicine	10,215	24	0.441	0.617	0.514
(l3-65)	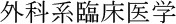	Clinical surgery	5,562	20	0.418	0.468	0.442
(l3-66)	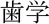	Dentistry	2,523	3	0.640	0.280	0.389
(l3-67)	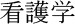	Nursing	158	2	0.737	0.443	0.553
Average			1,450.4	6.1	0.315	0.367	0.317

Here, the top three subject categories which have greater maximum *F*_1_-measure were “Mathematics” (l3-33), “Literature” (l3-21), and “Economics” (l3-28), whose pseudo average precision, recall, and maximum *F*_1_-measure were (0.734, 0.792, 0.762), (0.700, 0.683, 0.691), and (0.692, 0.622, 0.655), respectively. The number of WoS subject categories to get the maximum pseudo *F*_1_-measures were 4, 10, and 12, respectively. This means the corresponding WoS subject categories are much more relevant to the KAKENHI subject category. On the other hand, the bottom three subject categories which have less maximum *F*_1_-measure were “Cultural Assets Study” (l3-09), “Laboratory Animal Science” (l3-03), and “Genome Science” (l3-14), whose pseudo average precision, recall, and maximum *F*_1_-measure were (0.200, 0.036, 0.062), (0.059, 0.074, 0.066), and (0.040, 0.203, 0.067), respectively. The number of WoS subject categories to get the maximum pseudo *F*_1_-measures were 1, 1, and 3, respectively. This means the corresponding WoS subject categories are less relevant to the KAKENHI subject category. The overall pseudo average precision, recall, and maximum *F*_1_-measure were 0.315, 0.367, and 0.317, respectively. The number of WoS subject categories to get the maximum pseudo *F*_1_-measures ranged from 1 to 24 whose average is 6.1, which is rather small when compared with the maximum number 251. The cardinality of the KAKENHI subject categories ranged from 9 to 10,215 whose average is 1,450.4. The larger the cardinality is, the more reliable the measure is, because of law of large numbers.

#### Miscellaneous Considerations

Apart from the quantitative analysis mentioned previously, we set the threshold in the contingency table to ignore the relations between the 251 WoS subject categories and the 67 disciplines of the KAKENHI subject categories. Here, for every WoS subject category $O_{i}^{\left(1\right)}$, the number of relations with the KAKENHI subject categories $O_{j}^{\left(2\right)}$ is only at a maximum of 1–4. Moreover, when the recall rate for $O_{i}^{\left(1\right)}$ exceeds 0.5, we discontinued adding any relation.

We next verified all the correspondence between $O_{i}^{\left(1\right)}$ and $O_{j}^{\left(2\right)}$ by means of subject category keywords, specifically for subject categories with few evidence data. The cases are “Arts and Humanities,” “Music,” and “Religion,” among others of the $O_{i}^{\left(1\right)}$. This manual relation finding guarantees the existence of a finite cover by $O_{j}^{\left(2\right)}$.

Finally, we induced a correspondence between the 10 areas and 67 disciplines of the KAKENHI subject classification scheme and the 251 WoS subject categories, which are released in public^1^. A total of 324 relations are available in between the 10 areas of the KAKENHI subject classification scheme and the WoS subject categories, and 409 relations are present in between the 67 disciplines of the KAKENHI subject classification scheme and the WoS subject categories.

### Classification Results on the WoS Citation Database

With the correspondence, InCites™ preprocesses its internal database and provides the analysis functionality by using the KAKENHI subject classification scheme. The techniques used by the tool in providing the analysis function, its quantitative statistics, and user feedbacks of the function are discussed in the following sections.

#### KAKENHI Subject Categories of InCites™

**Figure 7:**
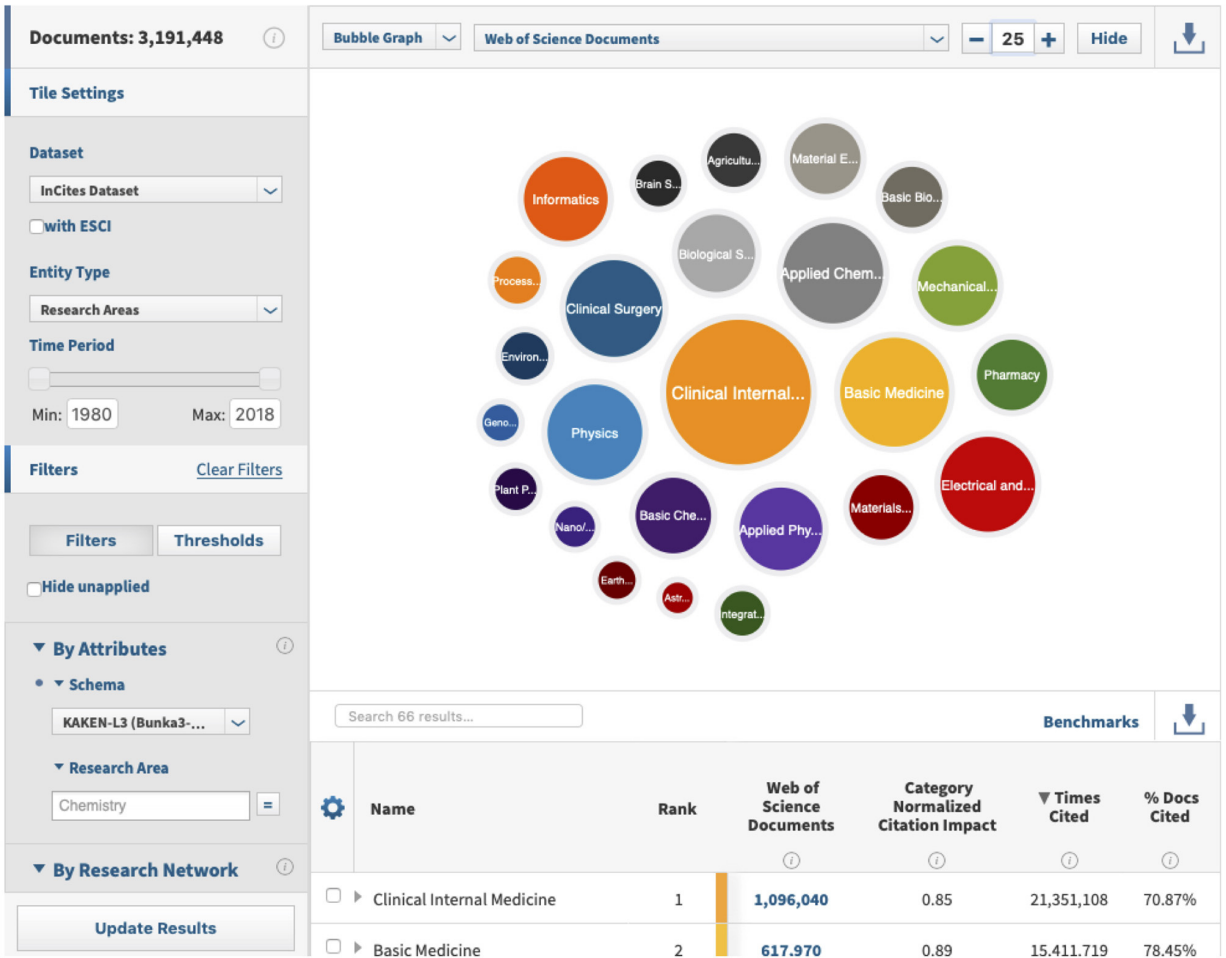
Example screen of InCites™, with the bubbles representing proportional numbers of articles classified using the KAKENHI subject categories.

InCites™ provides an analytical workbench on the WoS citation database. It preprocesses the database to demonstrate users' target entities such as people, organizations, regions, research areas, journals, books, conference proceedings, and funding agencies. Figure 7 shows an example screen presenting the article counts of Japanese authors based on the 67 disciplines of the KAKENHI subject classification scheme. The bubbles in the figure represent the top 25 proportional numbers of articles, each of which corresponds to the KAKENHI subject category. The total number of articles by the Japanese authors is 3,192,449 of the overall 58,395,008 articles published from 1980 to 2018. Among this Japanese authorship, the top or first KAKENHI subject category at the discipline level is “Clinical internal medicine,” with a total number of 1,096,040. The second and third are “Basic medicine” and “Applied chemistry,” with a total number of 617,970 and 526,139, respectively.

#### Article Counts Using the WoS and KAKENHI Subject Categories

**Figure 8:**
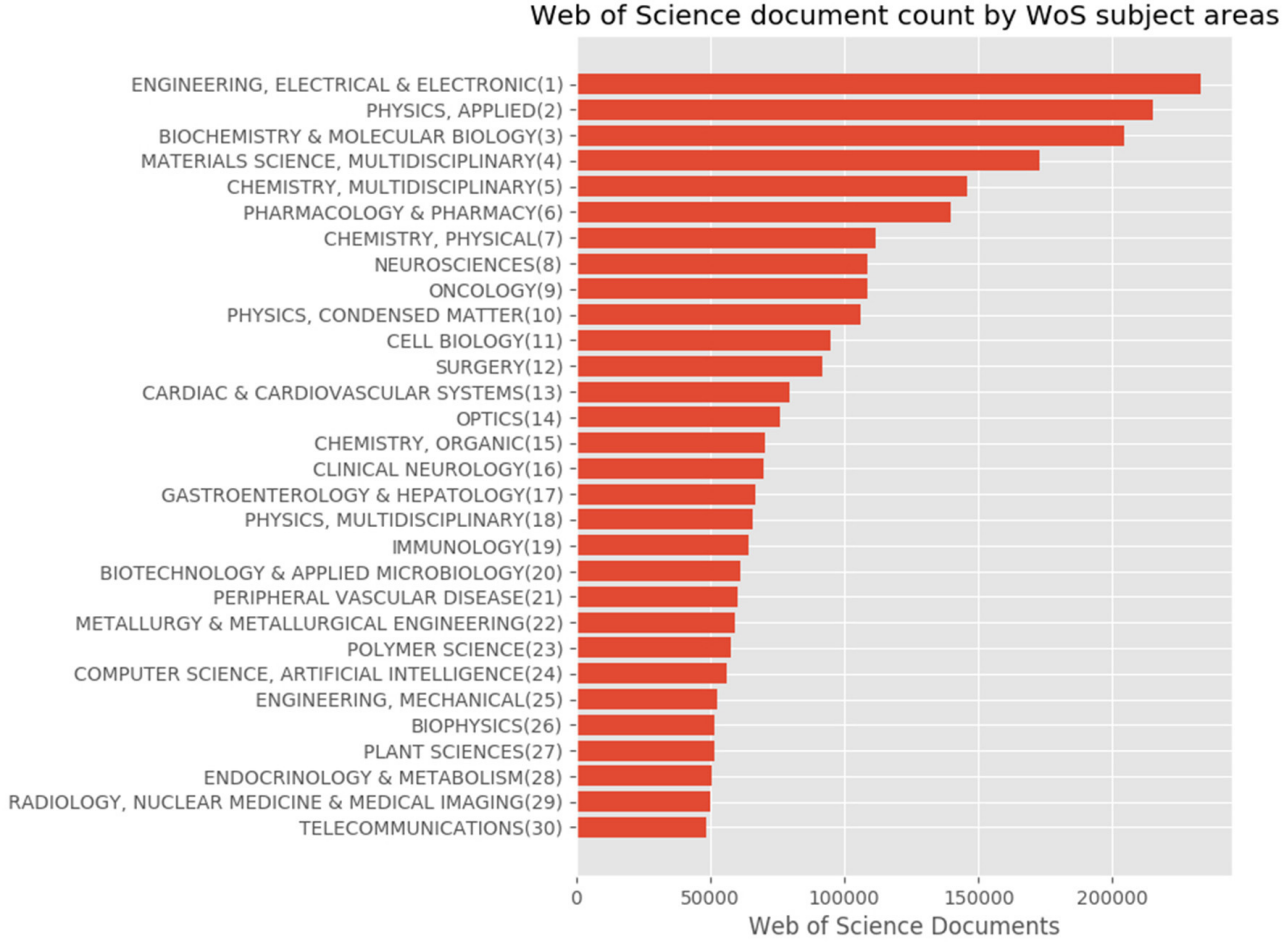
Top 30 subject distribution of Japanese authors' articles by using the WoS subject classification scheme.

**Figure 9:**
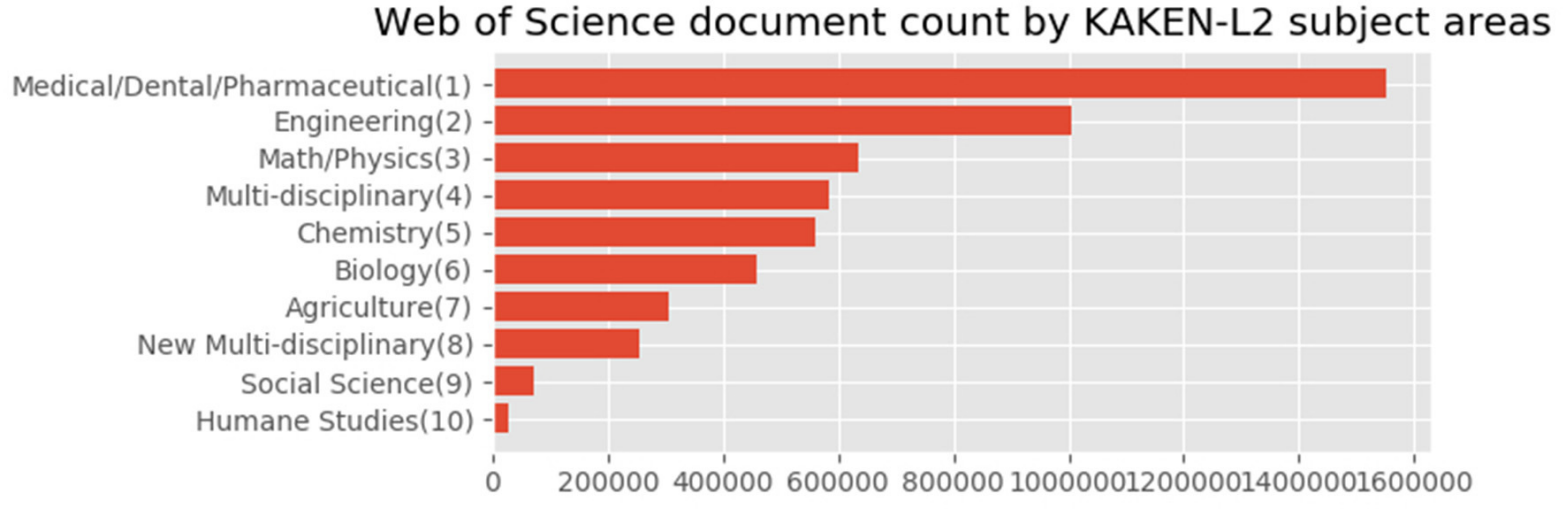
Entire subject distribution of Japanese authors' articles with the 10 areas level of the KAKENHI subject classification scheme.

**Figure 10:**
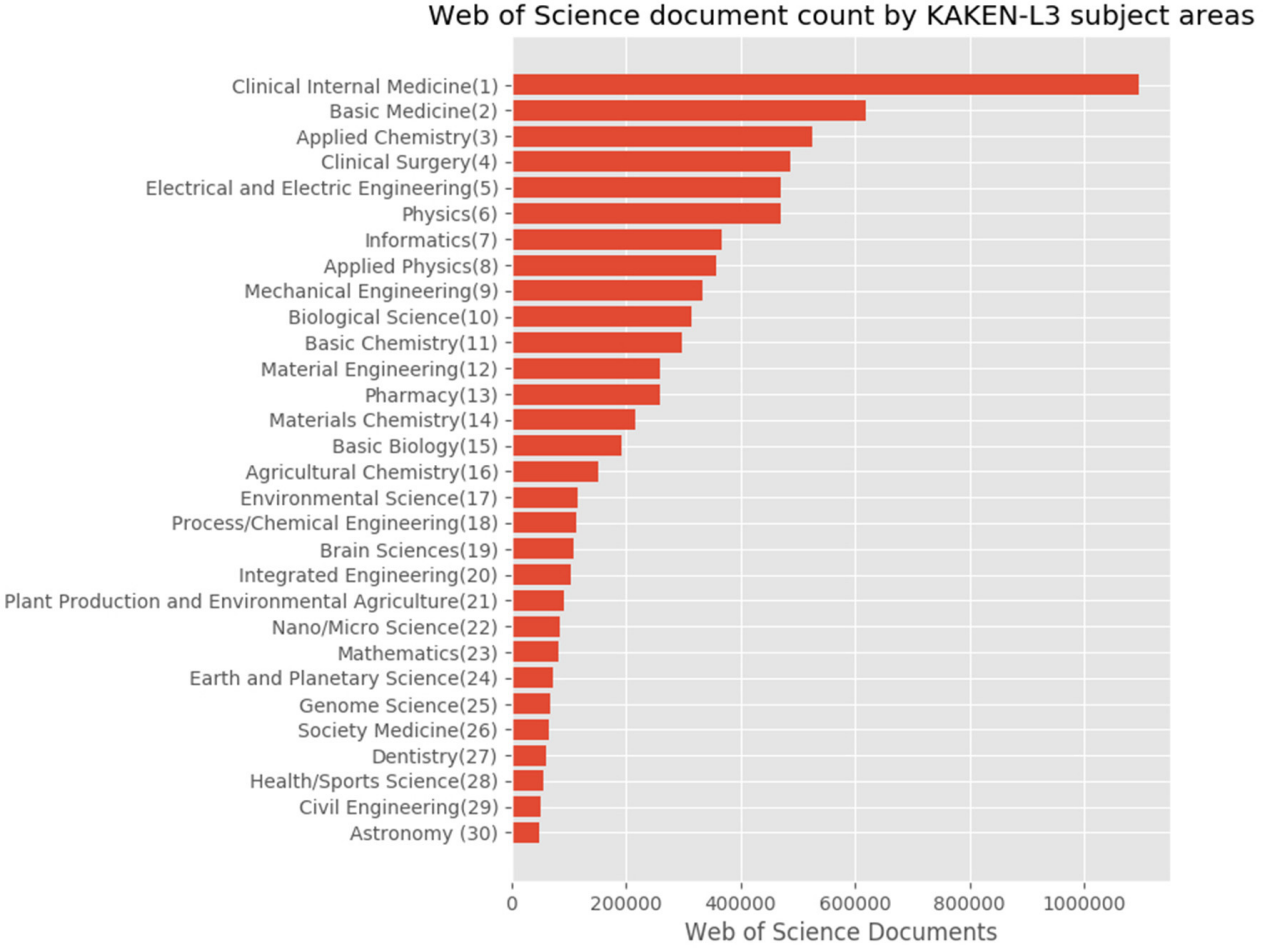
Top 30 subject distribution of Japanese authors' articles with the 67 disciplines level of the KAKENHI subject classification scheme.

For Japanese authors' articles, we compared the distributions using the subject classification schemes. We illustrated the proportions based on the statistics provided by the tool through the subject classification schemes shown in Figures 8–**10**.

Figure 8 shows the top 30 subject distribution of articles using the WoS subject classification scheme. At the top of the list are “Engineering, Electrical & Electronic,” “Physics, Applied,” “Biochemistry & Molecular Biology,” “Materials Science, Multidisciplinary,” and “Chemistry, Multidisciplinary,” among others. The distribution of the graph gradually declines similar to an inverse proportional graph.

Figure 9 shows the subject distribution of the same set of articles with the 10 areas level of the KAKENHI subject classification scheme. At the top of the list are “Medical/Dental/Pharmaceutical,” “Engineering,” “Math/Physics,” “Multidisciplinary,” and “Chemistry,” among others. The number of articles for the subject categories declines linearly rather than inversely. Figure 10 shows the top 30 subject distribution of articles by the 67 disciplines level of the KAKENHI subject classification scheme. At the top of the list are “Clinical Internal Medicine,” “Basic Medicine,” “Applied Chemistry,” “Clinical Surgery,” and “Electrical and Electric Engineering,” among others. The number of articles declines inversely. Unlike the original WoS subject categories, this statistical result provides a different impression that life sciences are the strongest among the others. However, the WoS subject classification scheme generates an impression that “Electrical/Electronic Engineering” and “Physics” are the strongest among the others.

#### User Feedback

In response to the KAKENHI subject classification scheme considering that a new function of InCites™ was released on April 2016, users in Japan were surveyed via an online questionnaire after a year: that is, in April 2017.

A total of 26 institutional users answered the questionnaire. They were mostly research administrators (RAs) and IR staff (Table 2).

**Table 2:** Users' role in their institutions.

**Users' role in the institution**	**Yes (multiple answers possible)**
RA (research administrator)	20
Administrator/officer	3
IR (institutional research) staff	5
Others	2

The questionnaire comprises 18 questions related to the subject classification schemes implemented in InCites™ and the attributes of users. An open-ended question was provided in the final part of the questionnaire.

**Figure 11:**
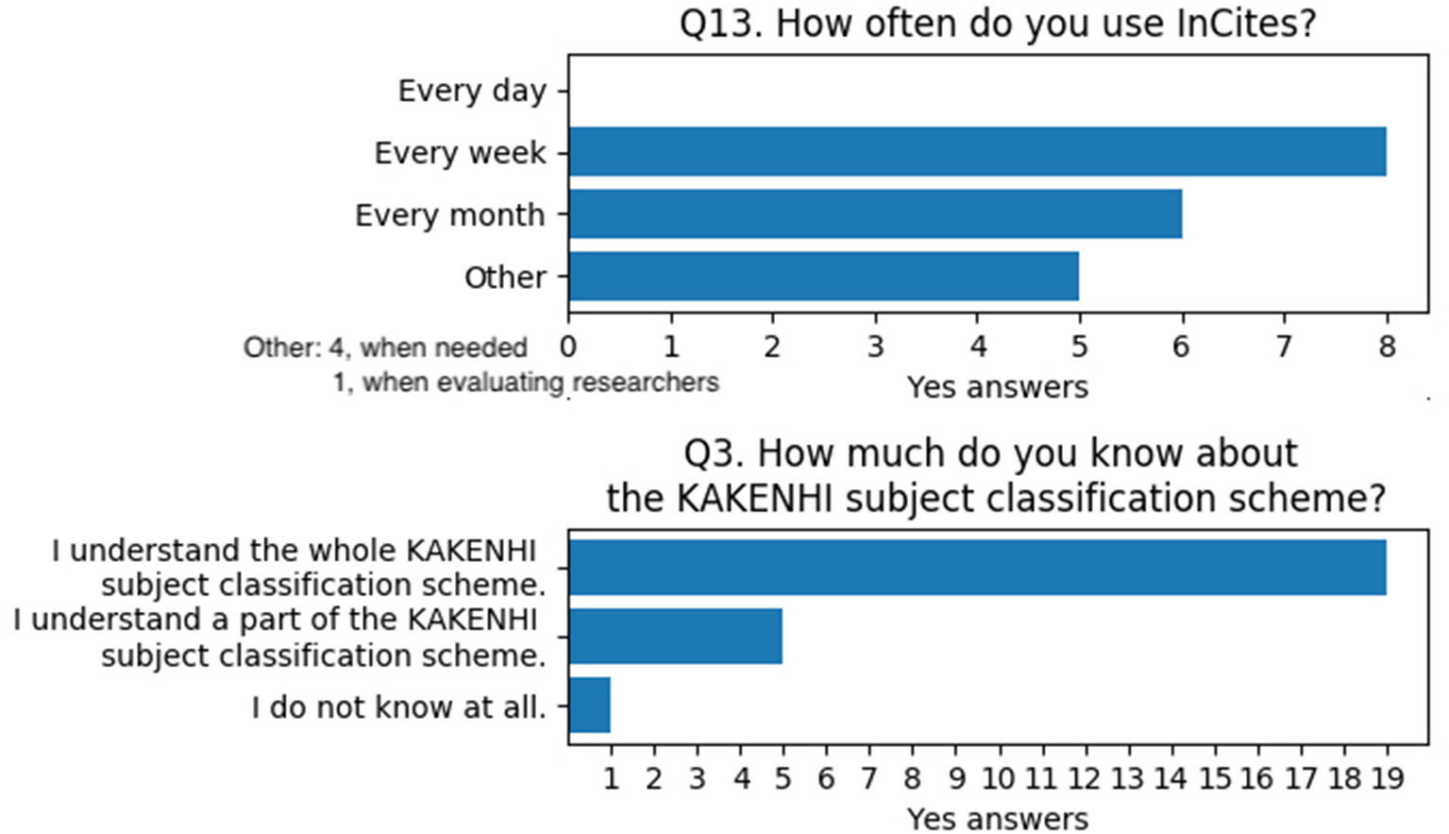
Questions and answers on users' degree of expertise for InCites™ and the KAKENHI subject classification scheme.

To determine the users' degree of expertise, Q13 and Q3 were prepared. Q13 asks how often users utilize InCites™, whereas Q3 asks how broad the users' knowledge is regarding the KAKENHI subject classification scheme. The results indicate that most of the users periodically utilize the tool in their work and have sufficient expertise on the KAKENHI subject classification scheme (Figure 11).

**Figure 12:**
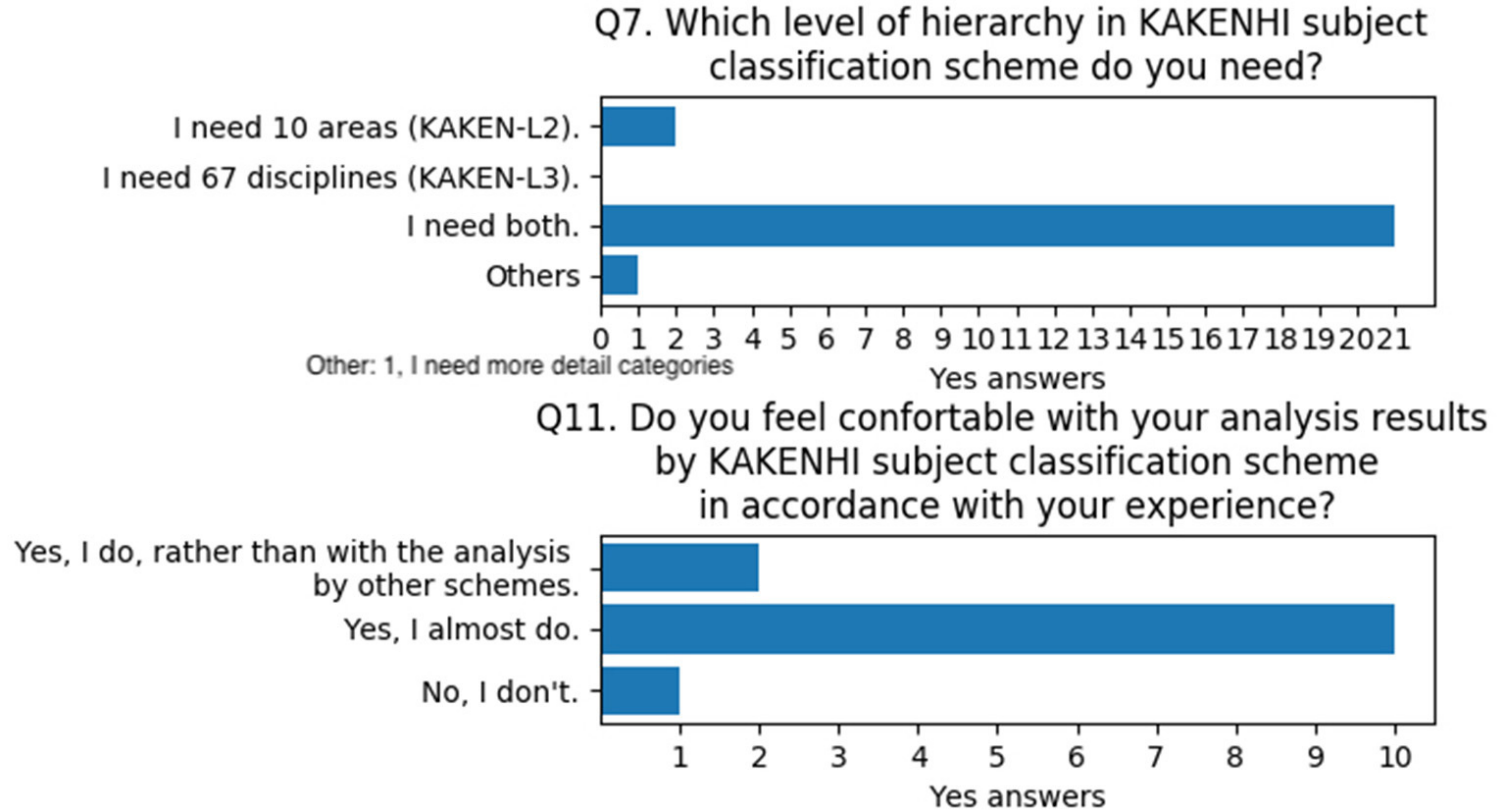
Questions and answers on the validity of the KAKENHI subject classification scheme.

With regard to the validity of the KAKENHI subject classification scheme, Q7 and Q11 were asked. Q7 investigates the necessary hierarchy level of the KAKENHI subject classification scheme. Q11 asks whether the users are comfortable with the analysis results when they use the KAKENHI subject classification scheme. The results of these questions indicate that users think they require both levels of hierarchy and are almost satisfied with the analysis results by using the KAKENHI subject classification scheme when compared with their experience with KAKENHI funding-related jobs (Figure 12).

In the section of the questionnaire where further comments on the new feature of the KAKENHI subject classification scheme were encouraged, several users insisted on its usefulness. Moreover, users stated that they needed the same subject categories for other services and wanted them updated. Their exact comments were as follows:

- “*I need the KAKENHI subject classification scheme in the Web of Science search service as well.”*
- “*I hope for updating the KAKENHI subject classification scheme to new* one *as possible. (It might be hard to catch up on updating it since it changes every year).”*
- “*Sixty-over categories of KAKENHI is not sufficient to relatively compare researches as much as ESI (22 only) and WoS (251*, four *times and more). And it may cause over-evaluation in comparison between research fields because the KAKENHI subject classification is made in a clock counter-like classification method. We need more accurate analysis of more concrete examples.”*

### Discussion

By analyzing the theory of our approach, that is, inducing a correspondence between the two subject classification schemes, we recognized its inherent limitation. The embedding subject classification scheme is unavoidably dependent on the original classification scheme. The topological space of the former is a subset of the topological space of the latter. However, we observed that each subject category of one scheme partially overlaps several subject categories of the other scheme based on the natural correlations between the subject categories of two subject classification schemes. No inclusion relationship exists between them. Therefore, the correspondence relations must be probabilistic.

In addition, we set strong assumptions on the relation among the research projects and journal articles in the research project database, in that they have similarities on the subject. However, the research projects and article outputs do have both similarities and differences on the subject. On the similarity side, we used the following procedure. A grants database reports that research projects produce outputs, that is, research articles. We focused on the subject classification scheme for the research projects and its relationship to a set of research articles. These articles were classified by using another subject classification scheme. Then, we compared the relationship of these two subject classification schemes. On the difference side, we have another issue. For example, the projects precede the articles. The time lag between project initiation and article outputs is observed, rendering a subject divergence or drift between them. Moreover, the projects tend to indicate the central concept using important keywords, allowing a subject diversification of the articles.

Nevertheless, users of InCites™ accepted the subject classification results. We assumed the following reasons. First, users might focus on the comparative analysis of bibliometrics based on the subject categories and not care about the specific case of articles. Second, they might require a rough quality of metrics during the evaluation stage. Metrics are the central limits of the quantitative attributes of a set of entities, which is the main indicator to be verified during the research evaluation.

Another advantage is that our approach requires less workload. In 2019, the number of WoS documents stored in InCites™ is 58,395,008, wherein the total number of journal titles amounts to 24,688. Thus, far, the possible targets for assigning subject categories are the WoS documents and journal titles. The journal titles include a set of documents. Furthermore, assigning subject categories to journal titles implies subsequently assigning them to the documents. In production, the WoS subject categories are primarily and exceptionally assigned to journal titles and documents in multidisciplinary journals. In our approach, we induced the correspondence between the WoS and KAKENHI subject classification schemes by using the KAKEN database. For the 251 WoS subject categories and 67 disciplines of the KAKENHI subject categories, the maximum relations in the correspondence are up to 16,817 (251 × 67). Regarding the 10 areas of KAKENHI subject categories, the maximum relations are up to 2,510 (251 × 10). The number for verifying the relations in our approach is overwhelmingly smaller than that of the original subject category manual assignment approach.

The evidence data are the contingency table whose sum of the frequency counts is 97,175. Specifically, this number is not sufficient for automatic decision making. When we assessed the correspondence between both subject classification schemes, the absence of relations is evident. The relations should be present in the literary meaning. Hence, manual handling was necessary for several subject categories. If the data size is sufficiently large, then we could predict the correspondence by using the data only.

## Conclusions and Future Work

In this study, we proposed an approach to apply a new subject classification scheme for a bibliographic database that is already classified by using a subject classification scheme. We also defined the subject classification model of the bibliographic database comprising a topological space. Then, we presented our approach based on the model, wherein forming a compact topological space is necessary for a novel subject classification scheme. To form the space, the correspondence between the two subject classification schemes by using the research project database was utilized as data.

We applied the approach to a practical example, that is, InCites™. This tool is used as a world proprietary benchmarking tool for research evaluation based on the WoS citation database to add the subject classification scheme of Japan's largest national grants KAKENHI. Finally, InCites™ provides a function of analysis by using the KAKENHI subject classification scheme. The survey indicates that users generally accept the new feature.

In future work, several aspects are necessary to improve the quality of the database and embed subject classification schemes by using effective and efficient automatic procedures. In real cases, there exists a more complex subject classification scheme. Our approach assumes that the subject classification schemes consist of a flat formation. For a complex classification scheme such as a hierarchical classification scheme, our approach should be extended to be applied to its character. Alternatively, multilabel learning is another possible method to aim at our goal. A comparative study is needed to qualify our method.

## Data Availability Statement

The datasets generated for this study are available on request to the corresponding author.

## Author Contributions

All authors listed have made a substantial, direct and intellectual contribution to the work, and approved it for publication.

### Conflict of Interest

The authors declare that this study received funding from Clarivate Analytics, Co., Ltd. The funder was not involved in the study design, collection, analysis, interpretation of data, the writing of this article or the decision to submit it for publication.

## Footnotes

^1^Clarivate Analytics. *KAKEN Category Scheme - InCites Help*. Available online at: http://help.prod-incites.com/inCites2Live/filterValuesGroup/researchAreaSchema/kaken.html (accessed February 21, 2019).
